# Quantifying ultra-rare pre-leukemic clones via targeted error-corrected sequencing

**DOI:** 10.1038/leu.2015.17

**Published:** 2015-02-20

**Authors:** A L Young, T N Wong, A E O Hughes, S E Heath, T J Ley, D C Link, T E Druley

**Affiliations:** 1 Department of Pediatrics, Division of Hematology and Oncology, Washington University School of Medicine, Saint Louis, MO, USA; 2 Center for Genome Sciences and Systems Biology, Washington University School of Medicine, Saint Louis, MO, USA; 3 Department of Medicine, Division of Oncology, Washington University School of Medicine, Saint Louis, MO, USA

The quantification of rare clonal and subclonal populations from a heterogeneous DNA sample has multiple clinical and research applications for the study and treatment of leukemia. Specifically, in the hematopoietic compartment, recent reports demonstrate the presence of subclonal variation in normal and malignant hematopoiesis,^1,2^ and leukemia is now recognized as an oligoclonal disease.^3^ Currently, clonal heterogeneity in leukemia is studied using next-generation sequencing (NGS) targeting subclone-specific mutations. With this method, detecting mutations at 2–5% variant allele fraction (VAF) requires costly and time-intensive deep resequencing and identifying lower frequency variants is impractical regardless of sequencing depth. Recently, various methods have been developed to circumvent the error rate of NGS.^4, 5^ These methods tag individual DNA molecules with unique oligonucleotide indexes, which enable error correction after sequencing.

Here we present a direct application of error-corrected sequencing (ECS) to study clonal heterogeneity during leukemogenesis and validate the accuracy of this method with a series of benchmarking experiments. Specifically, we demonstrate the ability of ECS to identify leukemia-associated mutations in banked pre-leukemic blood and bone marrow from patients with either therapy-related acute myeloid leukemia (t-AML) or therapy-related myelodysplastic syndrome (t-MDS). T-AML/t-MDS occurs in 1–10% of individuals who receive alkylator- or epipodophyllotoxin-based chemotherapy or radiation to treat a primary malignancy.^6^ For the seven individuals surveyed in this study, matched leukemia/normal whole-genome sequencing identified the t-AML/t-MDS-specific somatic mutations present at diagnosis. We applied our method for ECS to identify leukemia-specific mutations in four individuals from DNA extracted from blood and bone marrow samples collected years before diagnosis. In a separate study into the role of *TP53* mutations in t-AML/t-MDS leukemogenesis, this method was used to identify leukemia-associated mutations at low frequency in samples banked years before diagnosis.^7^ In two cases, subclones were identified below the 1% threshold of detection governed by conventional NGS. These results highlight the ability of targeted ECS to identify clinically silent single-nucleotide variations (SNVs).

We employed ECS by tagging individual DNA molecules with adapters containing 16 bp random oligonucleotide molecular indexes in a manner similar to other reports.^4, 5, 8^ Our implementation of ECS easily targets loci of interest by single or multiplex PCR and inserts seamlessly into the standard NGS library preparation (Supplementary Figure 1, Supplementary Methods). Our only deviations from the standard protocol are ligation of customized adapters containing random indexes instead of the manufacturer's supplied adapters and a quantitative PCR (qPCR) quantification step before sequencing (Supplementary Table 1). Following sequencing, sequence reads containing the same index and originating from the same molecule are grouped into read families. Sequencing errors are identified by comparing reads within a read family and removed to create an error-corrected consensus sequence (ECCS). We performed a dilution series experiment to assess bias during library preparation and determine the limit of detection for ECS. For this experiment, we spiked DNA from a t-AML sample into control human DNA, which was serially diluted over five orders of magnitude. The experiment was comprised of two technical replicates targeting two separate mutations (20 total independent libraries). The results demonstrate that ECS is quantitative to a VAF of 1:10 000 molecules and provides a highly reproducible digital readout of tumor DNA prevalence in a heterogeneous DNA sample (*r*^2^ of 0.9999 and 0.9991, Figures 1a and b). We next characterized the error profile based on the wild-type nucleotides included in the dilution series experiment. Variant identification using the ECCSs was 99% specific at a VAF of 0.0016 versus 0.0140 for deep sequencing alone (Figure 1c). We noticed that ECCS errors were heavily biased towards G to T transversions and to a lesser degree C to T transitions (Figure 1d, Supplementary Figure 2), as previously observed.^4, 9^ When separated by substitution type, variants identified from the ECCSs were 99% specific at a VAF of 0.0034 for G to T (C to A) mutations, 0.00020 for C to T (G to A) mutations and 0.000079 for the other eight possible substitutions. Although excess G to T mutations are a known consequence of DNA oxidation leading to 8-oxo-guanine conversion,^4^ the pre-treatment of samples with formamidopyrimidine-DNA glycosylase before PCR amplification did not appreciably improve the error profile of G to T mutations (Supplementary Figure 3).

As proof of principle, we applied ECS to study rare pre-leukemic clonal hematopoiesis in seven individuals who later developed t-AML/t-MDS. Leukemia/normal whole-genome sequencing at diagnosis was used to identify the leukemia-specific somatic mutations in each patient's malignancy (Supplementary Table 2). We applied targeted ECS to query these 18 different loci in 10 cryopreserved or formalin-fixed paraffin-embedded blood and bone marrow samples that were 9–22-year old and banked up to 12 years before diagnosis (Supplementary Table 3).

We generated ~25 Gb of 150 bp paired-end reads from six Illumina (San Diego, CA, USA) MiSeq runs. We targeted 1–7 somatic mutations per individual (25 mutations spanning 5.5 kb from 15 genes in total) and identified leukemia-specific subclonal populations in four individuals up to 12 years before diagnosis (Table 1). For each sequencing library, we tagged ~2.5 million locus-specific amplicons generated from genomic DNA using high-fidelity PCR with randomly indexed custom adapters. Sequencing errors were removed to create ECCSs as described above. Each ECCS was then aligned to the reference genome for variant calling (Supplementary Figure 1).

Using conventional deep sequencing, we detected t-AML/t-MDS-specific mutations in prior banked samples at variant allele fractions between 0.03 and 0.87 (data not shown). In one individual (UPN 684949), deep sequencing alone was insufficient to distinguish known *ASXL1* and *U2AF1* mutations from the sequencing errors in samples banked 5 and 3 years before t-MDS diagnosis, respectively (Figures 1e and f). However, ECS identified the L866* nonsense mutation in *ASXL1* at a VAF of 0.004 (Figure 1g) and the S34Y missense mutation in *U2AF1* at a VAF of 0.009 (Figure 1h). In addition, ECS was able to temporally quantify these mutations from three pre-t-MDS samples banked yearly from 3 to 5 years before diagnosis (Supplementary Figures 4 and 5). In two cases (UPN643006 and UPN942008), only a subset of the variants identified at diagnosis were present in the prior banked sample (Table 1). Specifically, in the UPN643006 sample, banked 12 years before diagnosis, a single-nucleotide deletion in *ASXL1* was present at VAF 0.03. But, the G to T substitution in *ASXL1*, CTT deletion in *GATA2* and G to T substitution in *U2AF1* were not detectable in this prior banked sample.

Here we present a practical and clinically oriented application for targeted error-corrected NGS utilizing single molecule indexing. This method easily integrates into existing NGS library preparation protocols and enables the quantification of previously undetectable mutations in heterogeneous DNA samples. The only modification to the standard NGS library preparation is the replacement of the stock adapters with our randomly indexed adapters and the addition of a qPCR step before sequencing. The qPCR step limits the number of molecules sequenced, ensuring adequate coverage for each read family. With these two modifications, we achieve highly specific detection for rare mutations. The bioinformatics analysis is straightforward and does not require proprietary algorithms or tools (Supplementary Methods). Our results highlight the ability of this method to identify rare subclonal populations in a heterogeneous biological sample. As applied to t-AML/t-MDS, we show these previously undetectable mutations are present years before diagnosis and fluctuate in prevalence over time.

A clinical application of ECS is to quantify minimal residual disease (MRD). As the genomic characterization of leukemia becomes more readily available, identifying causative genetic lesions and rare therapy-resistant subclones will become increasingly useful for risk stratification, therapeutic selection and disease monitoring. Already, whole-genome sequencing of AML has demonstrated that nearly every case of AML harbors one or more somatic SNVs.^10^ These SNVs are more reliable clonal markers of malignancy than cell surface markers, which can change over time. Leveraging this information, conventional NGS was implemented retrospectively to detect MRD harboring leukemia-specific insertions/deletions (indels) as rare as 0.00001 VAF in *NPM1*^11^ and 0.0001 VAF in *RUNX1*.^12^ This was possible because indels are only rarely generated erroneously by NGS. Unfortunately, measuring rare leukemia-associated substitutions is limited owing to the relatively high error profile of conventional NGS.^13^ However, ECS can achieve the 1:10 000 limit of detection featured by conventional MRD platforms.^14^ For patients whose leukemia lacks suitable markers for conventional MRD, ECS could offer an alternative with comparable sensitivity and specificity that is easy to implement in a clinical sequencing lab. Furthermore, the ability to multiplex targets for ECS enables the surveillance of known mutations and the simultaneous discovery of new somatic mutations. Ongoing work will directly compare gold-standard MRD methods with targeted ECS in patients with and without relapsed leukemia.

## Figures and Tables

**Figure 1:**
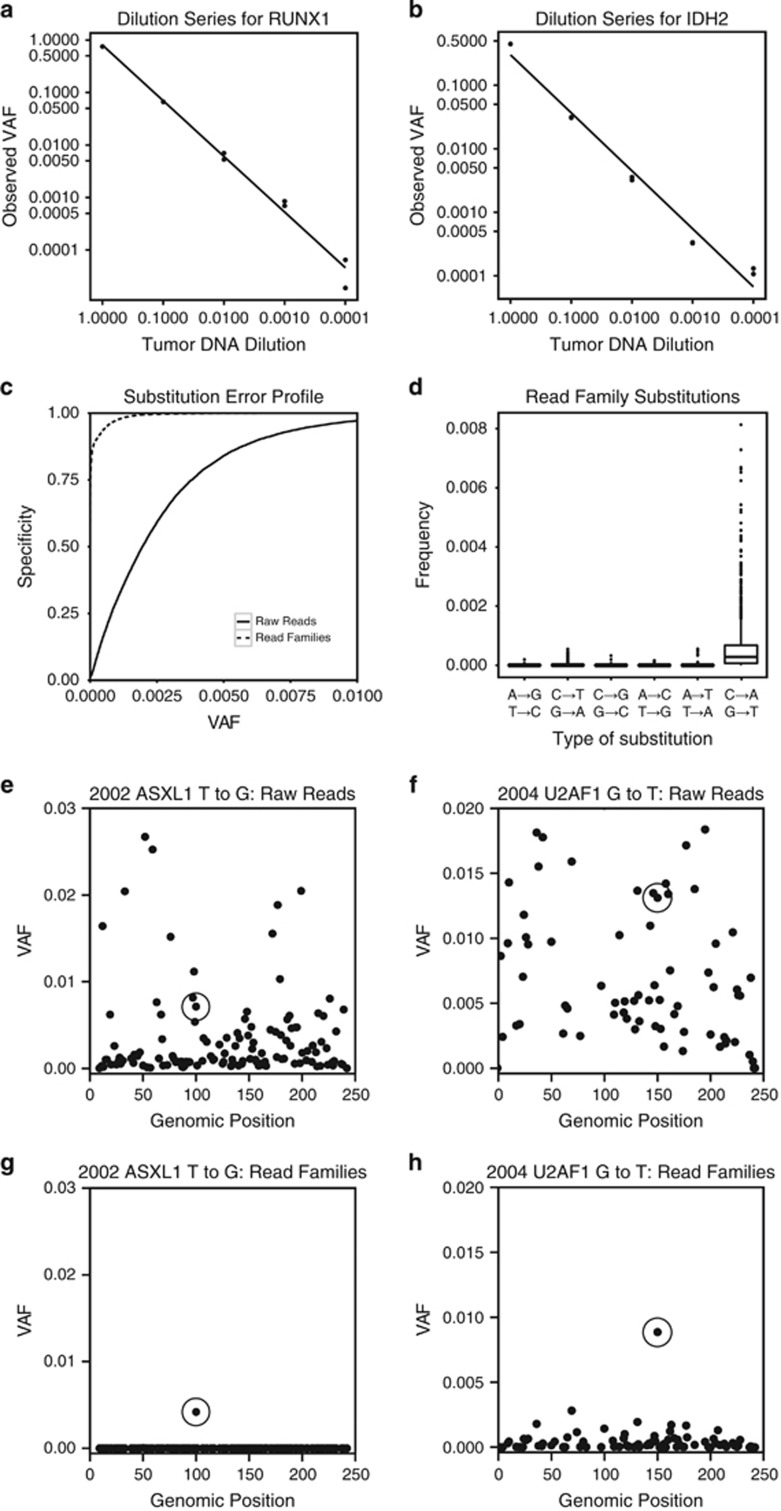
Benchmarking for ECS and the identification of rare pre-leukemic mutations. (**a**, **b**) DNA extracted from a diagnostic leukemia sample with known mutations in *RUNX1* (**a**) and *IDH2* (**b**) was serially diluted into non-cancer, unrelated human DNA. Two replicates were run per sample/dilution. The coefficient of determination (*r*^2^) between diluted tumor concentration in the sample and VAF in the generated read families was 0.9999 and 0.9991 for *RUNX1* and *IDH2*, respectively. (**c**) The VAF at every nucleotide not expected to contain mutations in the dilution series experiment were analyzed to determine the error profile of the error-corrected consensus sequences compared with conventional deep sequencing. A cumulative distribution function of VAF demonstrated a reduced error profile in read families relative to conventional deep sequenced reads. (**d**) The most frequent class of substitution seen in read families was in G to T (C to A) transversions, which was consistent with oxidative conversion of guanine to 8-oxo-guanine. (**e**, **f**) The leukemia-specific variants identified in *ASXL1* and *U2AF1* at diagnosis (circled) were not distinguishable from sequencing errors in the same substitution class by conventional deep sequencing. (**g**, **h**) Targeted error-corrected sequencing identified the *ASXL1* variant in the 2002 banked sample at 0.004 VAF and the *U2AF1* variant in the 2004 banked sample at 0.009 VAF.

**Table 1:** Patient-specific leukemia-associated somatic mutations identified by ECS

*UPN*	*Sample ID*	*Years prior*	*Gene*	*Chr*	*Position*	*Mut*	*Amino-acid change*	*Variant RFs*	*Reference RFs*	*VAF*
446294	75.02	1	OBSCN	1	228461129	A to G	H1857R	61 238	156 986	0.2806
			TP53	17	7578271	T to A	H193L	220 551	110 047	0.6671
499258	24.06	2	RUNX1	21	36252865	C to G	R139P	2	486 196	0
574214	26.04	7	DMD	X	32827676	G to A	R187*	7	199 945	0
643006	80.01	12	ASXL1	20	31022448	G to T	G645C	7	85 781	0.0001
			ASXL1	20	31022442	del G	G645fs	2 898	82 245	0.034
			GATA2	3	128200135	del CTT	K390in_fr_del	0	4 187	0
			U2AF1	21	44524456	G to T	S34Y	85	414 613	0.0002
684949	91.01	5	ASXL1	20	31023112	T to G	L866*	3 583	853 598	0.0042
			U2AF1	21	44524456	G to T	S34Y	545	514 410	0.0011
	92.02	4	ASXL1	20	31023112	T to G	L866*	54 074	535 976	0.0916
			U2AF1	21	44524456	G to T	S34Y	11 195	355 276	0.0305
	93.01	3	ASXL1	20	31023112	T to G	L866*	17 319	573 629	0.0293
			U2AF1	21	44524456	G to T	S34Y	827	92 104	0.0089
856024	30.02	1	S100A4	1	153517192	A to G	F27L	0	211 512	0
			IGSF8	1	160062252	G to A	P516S	0	22 614	0
			PLA2R1	2	160798389	A to G	L1431P	2	338 616	0
			POU3F2	6	99282794	C to A	S15R	8	201 240	0
			ANKRD18B	9	33524645	G to A	C53Y	7	214 836	0
			ESR2	14	64701847	G to A	A416V	10	135 861	0.0001
			FBN3	19	8155081	G to A	P2029L	0	152 304	0
942008	33.04	9	IDH2	15	90631934	C to T	R88Q	23 170	236 587	0.0892
			RUNX1	21	36231791	T to C	D171G	40	253 168	0.0002
	107.01	<1	IDH2	15	90631934	C to T	R88Q	138 180	161 371	0.4613
			RUNX1	21	36231791	T to C	D171G	368 438	50 796	0.8788

Abbreviations: ECS, error-corrected sequencing; RFs, read families; VAF, variant allele fraction. Two to seven mutations were queried per individual and the number of read families containing the variant allele or reference allele were reported and used to calculate the variant allele fraction.
