# Supplementary material for: Quantifying ultra-rare pre-leukemic clones via targeted error-corrected sequencing
Source: Leukemia. 2015 Feb 20;29(7):1608–11. doi: 10.1038/leu.2015.17 (PMC4497921; doi:10.1038/leu.2015.17)
Supplement: Supplementary Methods [file leu201517x1.pdf]

Supplementary Methods

Quantifying ultra-rare pre-leukemic clones via targeted error-corrected sequencing

Andrew L. Young[1,2], Terrence N. Wong[3], Andrew E. O. Hughes[1,2], Sharon E. Heath[3], Timothy J. Ley[3], Daniel C. Link[3], Todd E. Druley[1,2]

[1]Department of Pediatrics, Division of Hematology and Oncology; [2]Center for Genome Sciences and Systems Biology and [3]Department of Medicine, Division of Oncology, Washington University School of Medicine

Study Design

Blood and bone marrow samples from patients treated for t-AML/t-MDS at Washington University were banked or accessed following informed consent under Human Research Protection Protocol #201011766. Patients included in this study underwent matched leukemia and non-cancer (skin) whole genome sequencing on the Illumina HiSeq 2500 platform, which identified tumor-specific somatic coding mutations in leukemia samples. Our study focused on identifying these known mutations from matched blood or bone marrow samples banked 1-12 years prior to the initial diagnosis of t-AML/t-MDS.

Sample Preparation

Genomic DNA was generated from either FFPE or cryopreserved peripheral blood or bone marrow samples using the QIAamp DNA FFPE Tissue or DNA Mini Kit (Qiagen). PCR primers were designed using primer3[1] to amplify regions harboring individual leukemia-specific mutations from the banked biological samples (Supplementary Table 4). The concentration of each purified DNA sample was determined using the Qubit dsDNA HS Assay Kit (Life Technologies). Genomic DNA (400-800 ng) was amplified using the Q5 High-Fidelity 2X Master Mix (New England Biolabs) in a 25 uL reaction with 0.5 uM primers (Supplementary Figure 1a). The following conditions were used: 98C for 30s; 16-30 cycles of 98C for 10s, 62-72C (based on a separate optimization) for 30s and 72C for 30s; 72C for 2m; hold 10C. The PCR reactions were purified using the Agencourt AMPure XP (Beckman Coulter) bead-based protocol without modification.

For a few of the patient samples, the amount of input genomic DNA was limited. In these cases, modifications were made to the protocol to amplify multiple leukemia-specific mutations from the same biological sample (multiplex PCR). Patient-specific primers were pooled during a first round of PCR and amplified for roughly 16 cycles, similar to pre-amplification described in TAm-

Seq[2]. After purification the DNA was split into a single PCR reaction per patient-specific SNVs and amplified using only that specific primer pair, again for roughly 16 cycles. This allowed us to generate diverse amplicon pools for multiple loci using only 400-800 ng of starting DNA.

ECS Library Preparation

The concentration of the purified PCR products was measured using the Qubit dsDNA HS Assay Kit (Life Technologies). NGS libraries were prepared from 800 ng of amplicons for each sample/mutation using the Illumina TruSeq DNA Sample Preparation Kit (Illumina). We replaced the Illumina-provided Y-shaped adapters with custom adapters containing a random 16 base pair oligonucleotide index sequence (Supplementary Table 1). Adapters were diluted to 40 uM in Tris-EDTA with 5 nM NaCl and annealed using the following conditions: 95C for 5m then decreased by 1C every 30s to 4C. Aside from the custom adapters used for ligation, the library preparation protocol from Illumina was mostly unchanged (Supplementary Figure 1b). Enrichment for correctly ligated products was completed using a 50 uL Q5 PCR amplification with 2 uL of ligation product and 0.5 uM Illumina specific primers under the following conditions: 98C for 30s; 6 cycles of 98C for 10s, 57C for 30s and 72C for 30s; 72C for 2m; hold 10C The PCR reaction was purified using a modified Ampure bead cleanup, which increased the size range of purification to remove adapter dimers. 100 uL of beads were washed twice with ddH_2O to remove the stock poly-ethylene glycol (PEG) solution. The solution was replaced with 25.5 uL 50% wt/vol PEG (Sigma), 37.5 uL 5M NaCl and 37 uL ddH_2O. The PCR reaction was added to this solution and purified per the standard Ampure protocol.

Quantification by qPCR

We sought to generate read families from a single randomly-indexed molecule with roughly seven-fold coverage. Given the bandwidth of a single Illumina MiSeq run was roughly 15-18 million read pairs, we sought to generate sequencing libraries from roughly 2.5 million

molecules. To achieve this, we quantified the concentration of each library using the qPCR NGS Library Quantification Kit, Illumina GA (Agilent Technologies). Based on the measured concentration, each library was diluted to 0.4 pM such that a 10 uL volume of the diluted library would contain ~2.5 million molecules. The 10 uL aliquot of diluted sequencing library was then amplified for 16-20 cycles and purified with the same Q5 and modified Ampure bead protocol used for the previous enrichment PCR step. The final library was visualized on a 2% SYBR Safe gel (Life Technologies) and quantified using Qubit dsDNA HS Assay Kit. When multiplexing samples on a single lane of sequencing, individual sequencing libraries were combined in equimolar amounts after enrichment PCR and the pooled sample was diluted and quantified using qPCR as stated previously. However, we also found it possible to pool amplicons in equimolar amounts after the initial genomic DNA amplification and make a single sequencing library. Up to 7 different amplicons were multiplexed on a single MiSeq run. Multiplexing was only possible with mutations in different genes or within different exons of the same gene because the samples were demultiplexed by alignment.

Sequencing

Each library was sequenced on the Illumina MiSeq instrument as specified by the manufacturer (Supplementary Figure 1c). Approximately, 5-10% of PhiX control DNA was spiked into each sequencing experiment. Each completed sequencing run contained roughly 15-18M paired-end 150 bp reads. Raw sequence reads were aligned to the PhiX genome using Bowtie 2[3]. Sequence reads aligning to PhiX were removed from further analysis. The remaining sequence reads were aligned to UCSC hg19/GRCh37 using Bowtie 2 for comparison against error-corrected consensus sequences (ECCS) derived from read families (below).

Error Corrected Consensus Sequences

Sequence reads containing the same index sequence (originated from the same randomly-indexed molecule) were aligned to each other to generate read families in a fashion similar to previously published methods[4,5] (Supplementary Figure 1d). Previous studies used a minimum read family size of three[5]. We found using a more stringent cutoff of five reduced the error rate in the read families (Supplementary Figure 6). The median read family size was seven reads per index (Supplementary Figure 7). Paired-end reads within a read family were error corrected in a stepwise fashion (Supplementary Figure 1e). First, at every position, the nucleotides called by each sequence read were compared and a consensus nucleotide was called if there was at least 90% agreement between the reads. If there was less than 90% agreement, an N was called in the consensus sequence at that position. Errors that occurred during library preparation and sequencing were removed because they were not shared between different reads within a read family. Second, an ECCS was thrown out if less than 90% of the 300 nucleotides comprising the paired-end read were assigned a non-N nucleotide. These ECCSs were locally aligned to UCSC hg19/GRCh30 using Bowtie2[3] (Supplementary Figure 1f). The aligned ECCSs were processed with Mpileup[6] using the parameters *–BQ0 –d 10000000000000*. This removed the coverage thresholds to ensure that all of the pileup output was returned regardless of variant allele fraction (VAF) or coverage. Variant allele factions comprised of both the expected mutations and the background errors for each sample were visualized using IGV[7] and graphically represented using ggplot2[8]. Each known variant was plotted relative to the error-profile of that specific substitution class (e.g. an expected C to T transition was compared against the C to T error profile). Variants distinguishable from the noise for that specific error class and located at the expected position within the amplicon were called true positives. The threshold for calling true variants varied based on the error profile of that substitution class. Based on our benchmarking studies we were 99% specific to detect variants above 0.0034 VAF for G to T (C to A) substitutions, 0.00020 VAF for C to T (G to A) substitutions and 0.000079 VAF for the other eight possible substitutions.

References

1 Untergasser A, Cutcutache I, Koressaar T, Ye J, Faircloth BC, Remm M *et al.* Primer3--new capabilities and interfaces. *Nucleic Acids Res* 2012; **40**: e115.

2 Forshew T, Murtaza M, Parkinson C, Gale D, Tsui DWY, Kaper F *et al.* Noninvasive identification and monitoring of cancer mutations by targeted deep sequencing of plasma DNA. *Sci Transl Med* 2012; **4**: 136ra68.

3 Langmead B, Salzberg SL. Fast gapped-read alignment with Bowtie 2. *Nat Methods* 2012; **9**: 357–9.

4 Kinde I, Wu J, Papadopoulos N, Kinzler KW, Vogelstein B. Detection and quantification of rare mutations with massively parallel sequencing. *Proc Natl Acad Sci U S A* 2011; **108**: 9530–5.

5 Schmitt MW, Kennedy SR, Salk JJ, Fox EJ, Hiatt JB, Loeb L a. Detection of ultra-rare mutations by next-generation sequencing. *Proc Natl Acad Sci U S A* 2012; **109**: 14508–13.

6 Li H, Handsaker B, Wysoker A, Fennell T, Ruan J, Homer N *et al.* The Sequence Alignment/Map format and SAMtools. *Bioinformatics* 2009; **25**: 2078–9.

7 Thorvaldsdóttir H, Robinson JT, Mesirov JP. Integrative Genomics Viewer (IGV): high-performance genomics data visualization and exploration. *Brief Bioinform* 2013; **14**: 178–92.

8 Wickham H. *ggplot2*. Springer New York: New York, NY, 2009 doi:10.1007/978-0-387-98141-3.
