# Supplementary material for: Quantifying ultra-rare pre-leukemic clones via targeted error-corrected sequencing
Source: Leukemia. 2015 Feb 20;29(7):1608–11. doi: 10.1038/leu.2015.17 (PMC4497921; doi:10.1038/leu.2015.17)
Supplement: Supplementary Tables and Figures [file leu201517x2.pdf]

Supplementary Tables and Figures

Quantifying ultra-rare pre-leukemic clones via targeted error-corrected sequencing

Andrew L. Young[1,2], Terrence N. Wong[3], Andrew E. O. Hughes[1,2], Sharon E. Heath[3], Timothy J. Ley[3], Daniel C. Link[3], Todd E. Druley[1,2]

[1]Department of Pediatrics, Division of Hematology and Oncology; [2]Center for Genome Sciences and Systems Biology and [3]Department of Medicine, Division of Oncology, Washington University School of Medicine

Supplementary Figure 1. Error-corrected sequencing workflow. Schematic depiction of library preparation and bioinformatics analysis for generating read families and error-corrected consensus sequences.

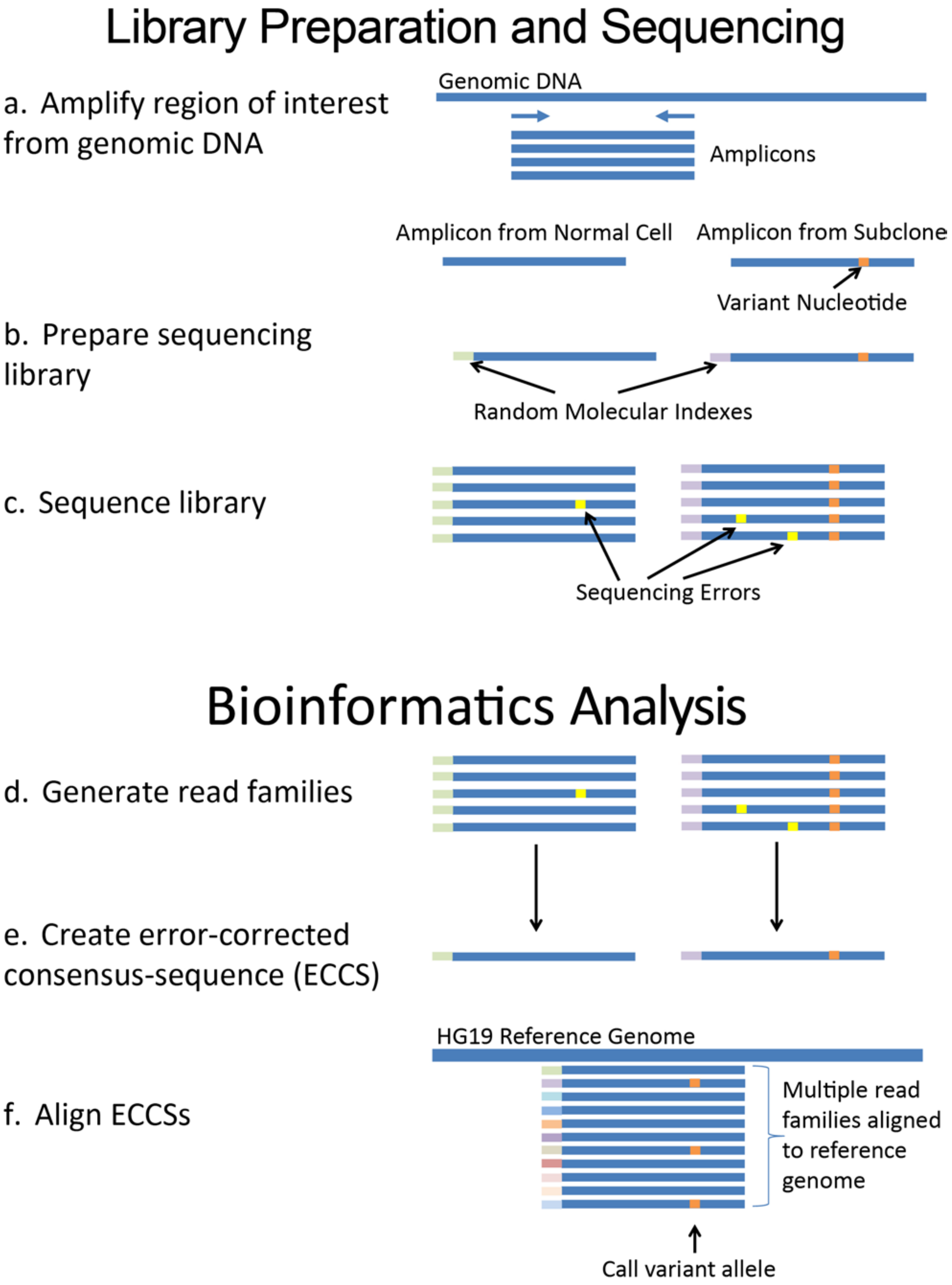

Supplementary Figure 2. Cumulative distribution function of the error profile comparing ECS to conventional deep sequencing. The variant allele fraction for each non-variant position covered in the dilution series experiment was sorted and plotted cumulatively. The variant allele fractions of errors were higher in every nucleotide covered across all substitution types for the raw sequenced reads compared the error-corrected consensus sequences generated from read families.

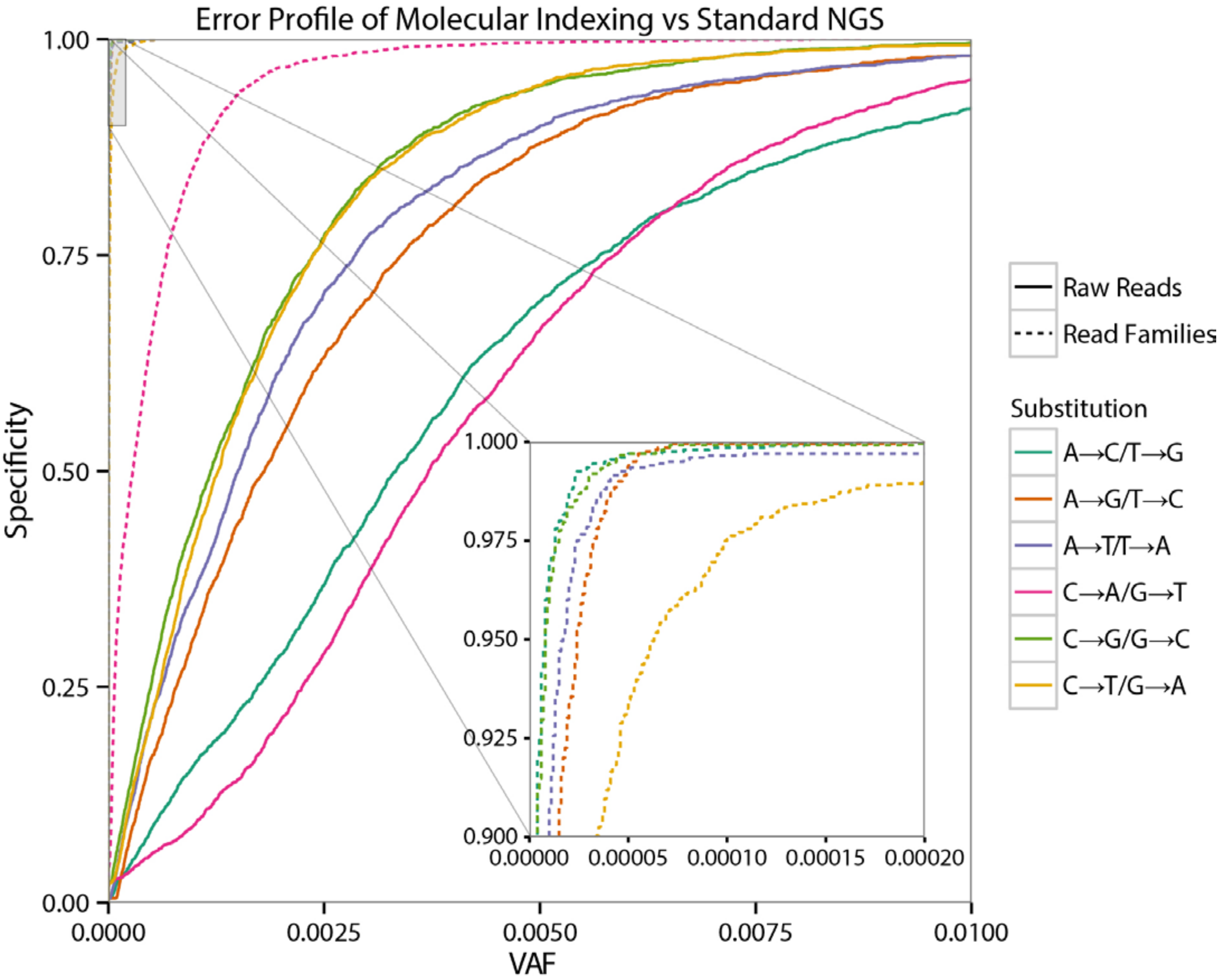

Supplementary Figure 3. Cumulative distribution function of read family error profile per specific substitution type with and without FPG pretreatment. The error profile of G to T (C to A) substitutions, consistent with guanine oxidation to 8-oxo guanine, was higher than the other classes of mutations. The C to T (G to A) substitutions, consistent with cytosine deamination to uracil, was visible just over the error profile for the remaining 8 types of substitutions (inset). FPG pretreatment did not appreciably change the error profile.

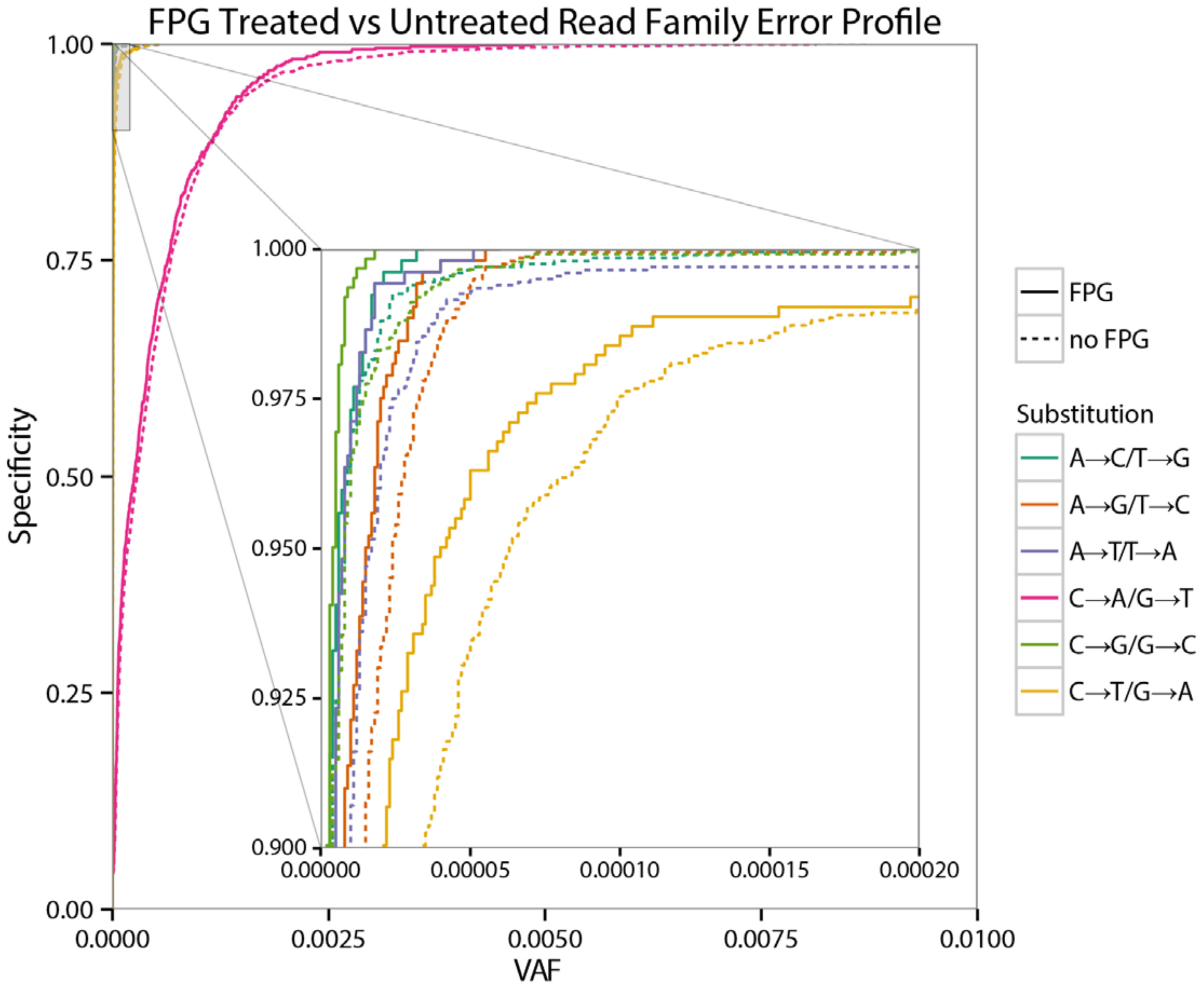

Supplementary Figure 4. *ASXL1* mutations over time in UPN684949. Formalin-fixed paraffin-embedded bone marrow samples were banked over three years from this individual. Conventional deep sequencing (a-c) only distinguished the *ASXL1* variant from the T to G sequencing errors in the 2003 banked sample at 0.097 VAF. Correcting the sequencing errors with ECS identified the *ASXL1* variant at 0.0042 VAF in 2002 (d), 0.092 VAF in 2003 (e) and 0.029 VAF in 2004 (f).

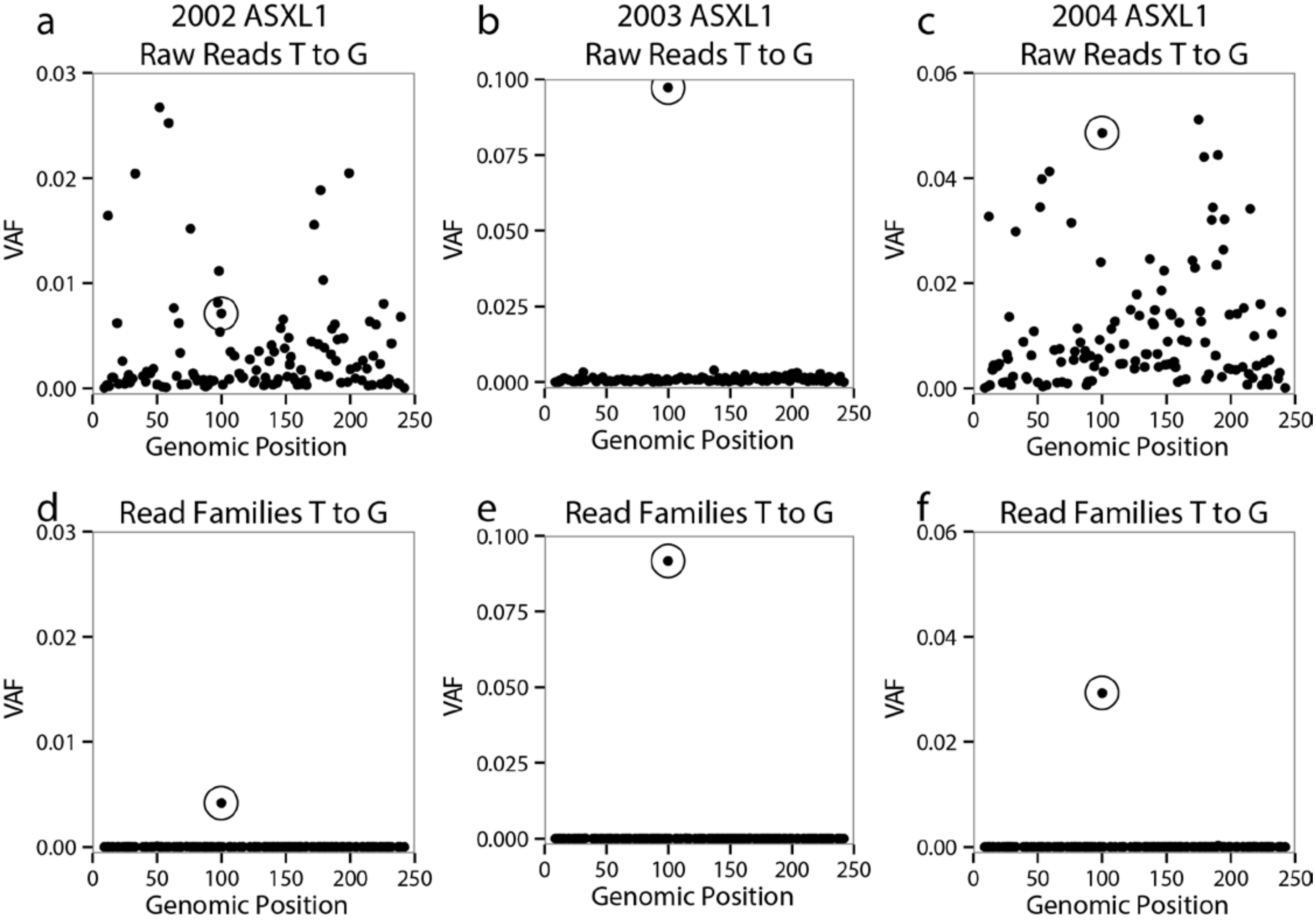

Supplementary Figure 5. *U2AF1* mutations over time in UPN684949. Formalin-fixed paraffin-embedded bone marrow samples were banked over three years from this individual. Conventional deep sequencing (a-c) only distinguished the *U2AF1* variant from the G to T sequencing errors in the 2003 banked sample at 0.036 VAF. Correcting the sequencing errors with molecular indexing did not identify the *U2AF1* variant in 2002 (d), but did identify the *U2AF1* variant at 0.031 VAF in 2003 (e) and 0.0089 VAF in 2004 (f).

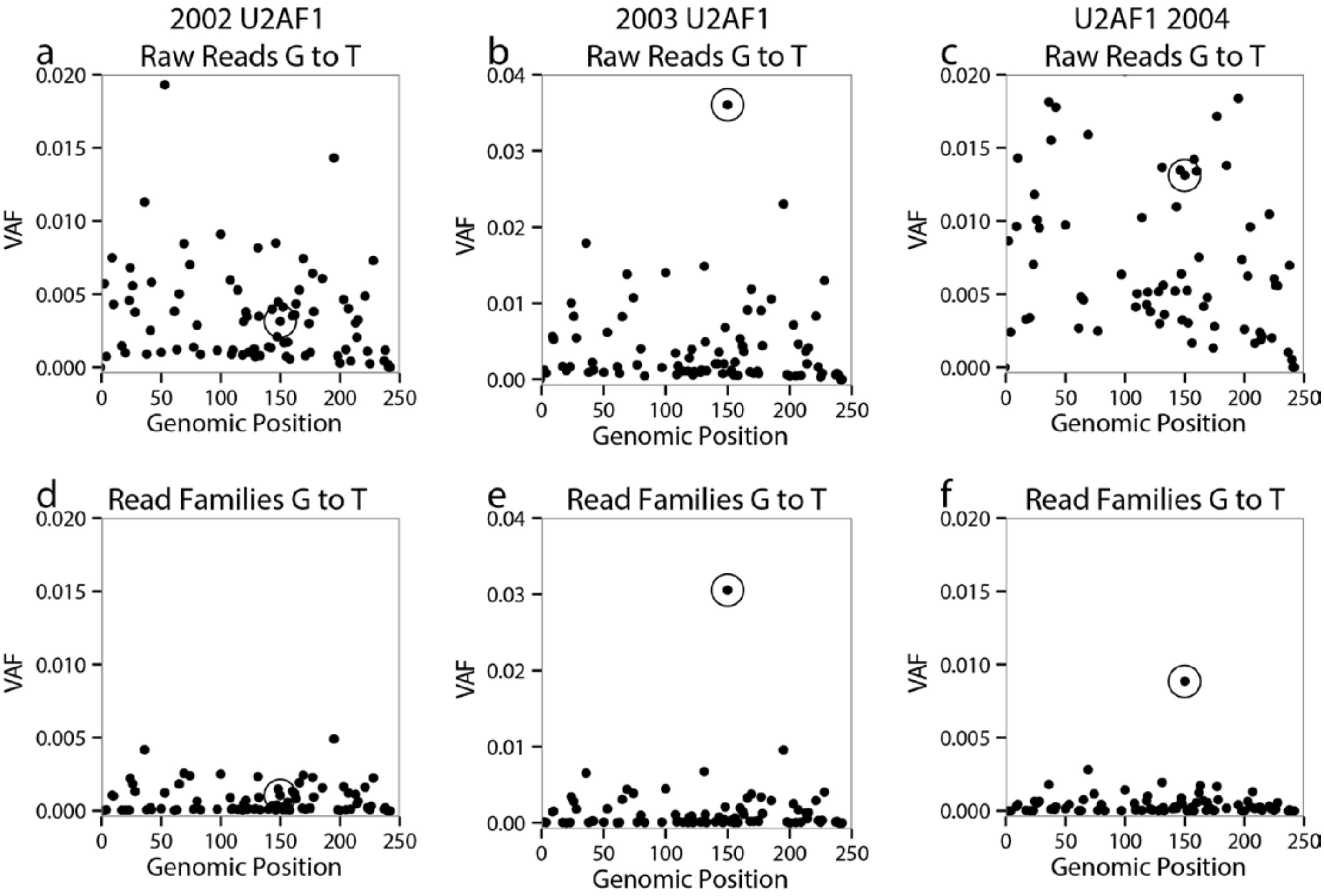

Supplementary Figure 6. Error profile observed with increased read family size. Read families generated with 3x or greater coverage (solid line) had a higher cumulative distribution of erroneous substitutions called compared to read families with 5x or greater coverage (dotted line).

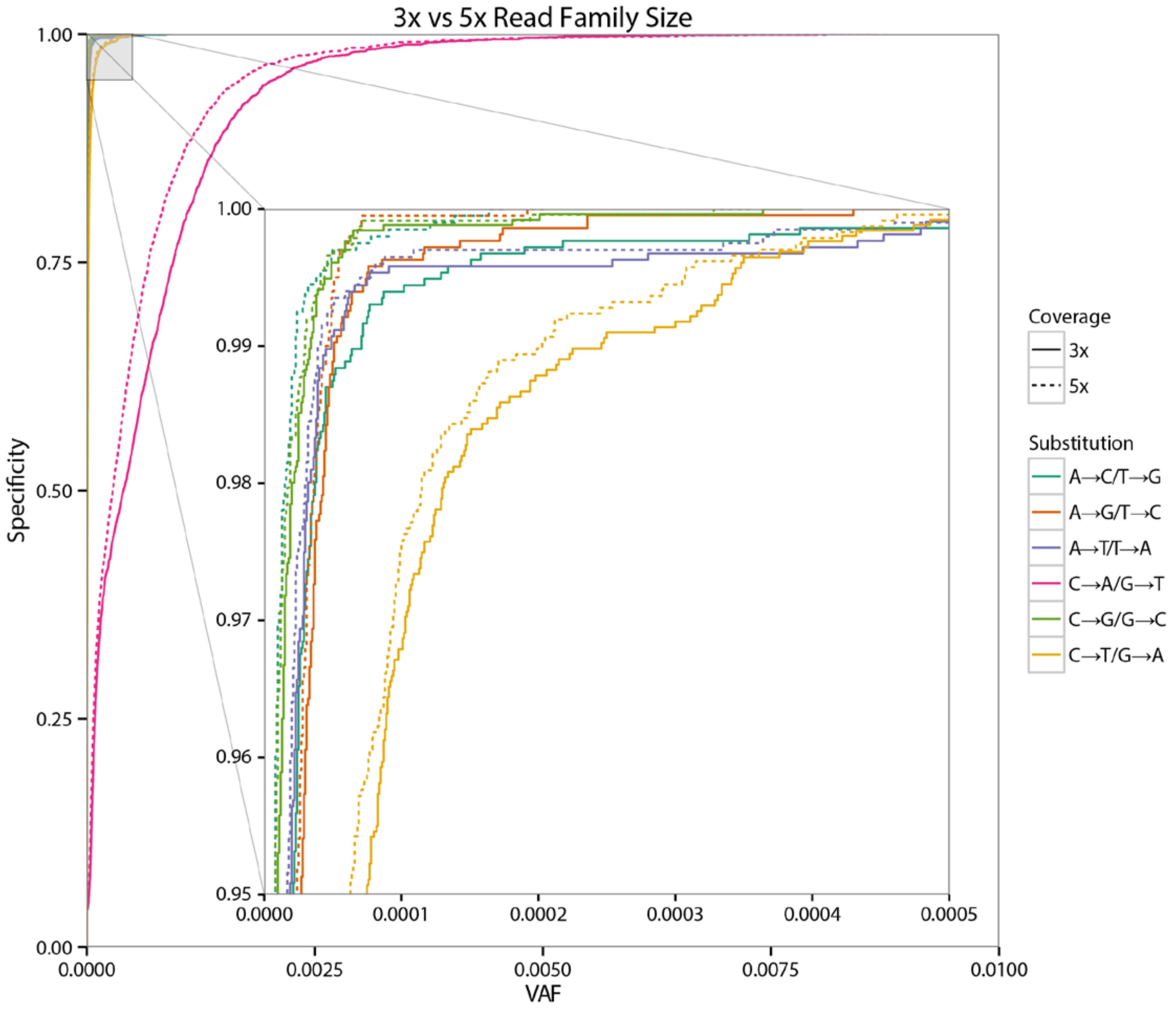

Supplementary Figure 7. Representative distribution of read family size. Singletons represent index sequences containing a sequencing error. Excluding singletons, the median read family size was 7x (mean 7.4x). Only read families with 5-20 reads were included in ECS analysis.

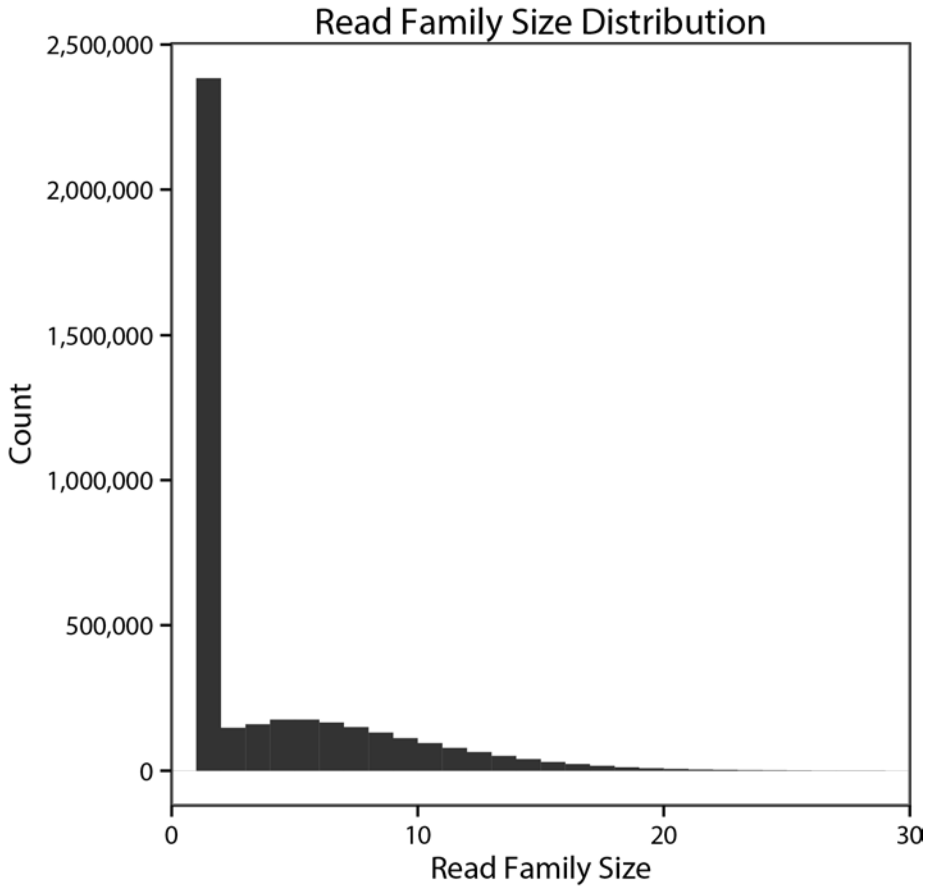

Supplementary Table 1. Random 16-mer molecular indexed adapters. The terminal 5-prime phosphorylation on complementary adapter sequence was used to improve ligation efficiency (*****).

Label	Sequence
16N Index Adapter	AGACGGCATACGAGATNNNNNNNNNNNNNNNNGTGACTGGAGTTCAGACGTGTGCTCTTCCGATCT
Complementary Adapter	*GATCGGAAGAGCGTCGTGTAGGGAAAGAGTGTAGATCTCGGTGGTCGCCGTATCATT

Supplementary Table 2. Whole-genome sequencing of diagnosis t-AML/t-MDS samples.

UPN	Gene	Chr	Position	Mutation	AA Change	Reference Reads	Variant Reads	VAF
446294	OBSCN	1	228461129	A to G	H1857R	3	5	0.63
	TP53	17	7578271	T to A	H193L	79	106	0.57
499258	RUNX1	21	36252865	C to G	R139P	122	17	0.12
574214	DMD	X	32827676	G to A	R187*	103	73	0.41
643006	ASXL1	20	31022448	G to T	G645C	36	32	0.47
	ASXL1	20	31022442	del G	G645fs	33	32	0.49
	GATA2	3	128200135	del CTT	K390in_frame_del	8	10	0.56
	U2AF1	21	44524456	G to T	S34Y	24	27	0.53
684949	ASXL1	20	31023112	T to G	L866*	75	14	0.16
	U2AF1	21	44524456	G to T	S34Y	57	9	0.14
856024	S100A4	1	153517192	A to G	F27L	103	48	0.32
	IGSF8	1	160062252	G to A	P516S	28	42	0.60
	PLA2R1	2	160798389	A to G	L1431P	45	33	0.42
	POU3F2	6	99282794	C to A	S15R	15	15	0.50
	ANKRD18B	9	33524645	G to A	C53Y	26	20	0.43
	ESR2	14	64701847	G to A	A416V	40	22	0.35
	FBN3	19	8155081	G to A	P2029L	54	38	0.41
942008	IDH2	15	90631934	C to T	R88Q	10	10	0.50
	RUNX1	21	36231791	T to C	D171G	15	35	0.70

Supplementary Table 3. Summary of patient information. The type of primary malignancy, the date of primary malignancy diagnosis, the date and type of blood/bone marrow banked prior to t-AML/t-MDS diagnosis and the date of t-AML/t-MDS diagnosis are included in the table below. At t-AML/t-MDS diagnosis, tumor/normal whole genome sequencing identified leukemia-specific mutations. Some of the prior banked blood/bone marrow samples showed evidence of subclonal populations harboring those leukemia-specific mutations before the clinical detection of disease.

UPN	Primary Malignancy Diagnosis	Date Primary Malignancy	Banked Samples	Banking Type	Date Banked	t-AML/t-MDS Diagnosis	Evidence of Pre-Leukemic Subclones
446294	Breast cancer	2002	75.02	FFPE	07/2005	2006 (t-MDS)	Yes
499258	Hodgkin's lymphoma	1998	24.06	Cryo	02/2002	2004 (t-MDS)	No
574214	Breast cancer	1998	26.04	Cryo	01/2000	2007 (t-MDS)	No
643006	AML	1989	80.01	FFPE	04/1992	2004 (t-MDS)	Yes
684949	CLL	09/1991	91.01	FFPE	11/2002	2007 (t-MDS)	Yes
			92.02	FFPE	09/2003		Yes
			93.01	FFPE	10/2004		Yes
856024	NHL	11/2004	30.02	Cryo	03/2005	2006 (t-AML)	No
942008	NHL	08/1992	33.04	Cryo	09/1996	2005 (t-AML)	Yes
			107.01	FFPE	11/2005		Yes

Supplementary Table 4. Primers targeting leukemia-specific variants. Primer sequences used to generate variant-specific amplicons from banked genomic DNA samples.

UPN	Gene	FWD Primer	Reverse Primer
446294	OBSCN	GGAGCCTCTGACCCTGCATCCCTCC	CCCGCCTCACAGCTGTACTCCCCAG
	TP53	AGACCTCAGGCGGCTCATAGGGCAC	GGGGCTGGAGAGACGACAGGGCTG
499258	RUNX1	TCACTAGAATTTTGAAATGTGGGTTTGTTGCC	GCACTCTGGTCACTGTGATGGCTGGC
574214	DMD	GGCGATGTTGAATGCATGTTCCAGT	AGGACTATGGGCATTGGTTGTCAAT
643006	ASXL1	GGACCCTCGCAGACATTAAAGCCCGT	GCCTCACCACCATCACCACTGCTGC
	GATA2	CCACAGGTGCCATGTGTCCAGCCAG	CTGTGGCGGGGTGGGAGGAATGTTG
	U2AF1	TGAACACAAATGGAAAATACAACTACGAGAGAAAA	CCCAGCAAAATAATCAGCTCTCATTTTCCC
684949	ASXL1	CACTATGAAGGATCCTGTAAATGTGACCCC	TGGTTTGGGCTGTTTCACTACCTCA
	U2AF1	TGAACACAAATGGAAAATACAACTACGAGAGAAAA	CCCAGCAAAATAATCAGCTCTCATTTTCCC
856024	S100A4	CCACGTGGGGACTCACTCAGGCA	AATAAGACGGTCTCTGTGCCTCCTG
	IGSF8	TGGTACACGCCTTCATCCTCGGG	GCTCAGCTCTGTCCCTGCCCAGCT
	PLA2R1	ACCCTGGTGTCTGTGGCATTCTCTG	AGTCACAGCATCATTCCTCTTGCGGT
	POU3F2	CAAATGCGCGGCTCCTTTAACCGGA	GCGTGGCTGAGCGGGTGTCC
	ANKRD18B	TACCACATTCGGGACTGGGAACTGC	CTCCCAGGGTCCCGGCGAACTCC
	ESR2	TGGCAATCACCCAAACCAAAGCATCGGT	AACCCAGATCACCTCGGAGCAGGCG
	FBN3	GGGGACACAGTTCGCAGGGGTC	GACTGGGGTGCGGGAGGTCACAGG
942008	IDH2	GGCGTGCCTGCCAATGGTGATGGG	CCGTCTGGCTGTGTTGTTGCTTGGGG
	RUNX1	ACATGGTCCCTGAGTATACCAGCCT	GGCCACCAACCTCATTCTGTTTTGT
